# Cancer risk at low doses of ionizing radiation: artificial neural networks inference from atomic bomb survivors

**DOI:** 10.1093/jrr/rrt133

**Published:** 2013-12-22

**Authors:** Masao S. Sasaki, Akira Tachibana, Shunichi Takeda

**Affiliations:** 1 Kyoto University, 17-12 Shironosato, Nagaokakyo-shi, Kyoto 617-0835, Japan; 2 Department of Biology, Faculty of Science, Ibaraki University, Bunkyo 2-1-1, Mito, Ibaraki 310-8512, Japan; 3 Department of Radiation Genetics, Graduate School of Medicine, Kyoto University, Yoshida-konoecho, Sakyo-ku, Kyoto 606-8501, Japan

**Keywords:** cancer risk, low-dose radiation, A-bomb survivors, artificial neural networks, integrate-and-fire model, DSB repair pathway choice

## Abstract

Cancer risk at low doses of ionizing radiation remains poorly defined because of ambiguity in the quantitative link to doses below 0.2 Sv in atomic bomb survivors in Hiroshima and Nagasaki arising from limitations in the statistical power and information available on overall radiation dose. To deal with these difficulties, a novel nonparametric statistics based on the ‘integrate-and-fire’ algorithm of artificial neural networks was developed and tested in cancer databases established by the Radiation Effects Research Foundation. The analysis revealed unique features at low doses that could not be accounted for by nominal exposure dose, including (i) the presence of a threshold that varied with organ, gender and age at exposure, and (ii) a small but significant bumping increase in cancer risk at low doses in Nagasaki that probably reflects internal exposure to ^239^Pu. The threshold was distinct from the canonical definition of zero effect in that it was manifested as negative excess relative risk, or suppression of background cancer rates. Such a unique tissue response at low doses of radiation exposure has been implicated in the context of the molecular basis of radiation–environment interplay in favor of recently emerging experimental evidence on DNA double-strand break repair pathway choice and its epigenetic memory by histone marking.

## INTRODUCTION

The Life-Span Study (LSS) of the Radiation Effects Research Foundation (RERF), which involves a long-term follow-up of atomic bomb (A-bomb) survivors in Hiroshima and Nagasaki, has provided fundamental information on cancer risk in humans following exposure to ionizing radiation. The dose dependency of cancer risk for survivors exposed to moderate-to-high radiation doses has been unequivocally established [1, 2]. However, statistical properties still limit the analysis at lower doses (<0.2 Sv) where the dose–response is imprecise, and its resolution is hampered by limited statistical power. In this circumstance, despite its unproven assumptions, a linear non-threshold (LNT) model has been pragmatically adopted in the setting of radiation protection standards [3, 4]. However, the projection of the dose–response to publicly or occupationally relevant low doses has long been a matter of intense debate [5, 6]. Limitations are also due to uncertainties with regard to radiation doses that are accountable for residual radiation, radionuclide fallout and internal exposure. Furthermore, some unexplained transient elevations of cancer risk have been noted in the 0.15–0.3 Sv dose range [7], which cannot simply be described by current radiobiological knowledge on issues such as genomic instability, the bystander effect, adaptive responses or heterogeneous genetic susceptibility [5, 8].

To deal with these complications, we developed here a novel non-parametric statistical procedure based on the noise cancellation process of the artificial neural networks (ANN) theorem, and evaluated cancer risk in A-bomb survivors exposed to low doses of radiation. ANN is a mathematical construct modeled on the ‘integrate-and-fire’ excitation response of neurons [9, 10], in which the firing of an action potential occurs when the membrane potential arising from the accumulation of small, depolarizing input signals reaches a threshold. Apart from its application to neurophysiology, the mathematical algorithm of the threshold process has been used extensively in a variety of information technology fields, including image processing, robotics, etc. [11, 12]. The rationale behind the use of the ANN theorem in cancer statistics is that, if we consider the cancer rate at given radiation dose as an input variable, the same algorithm would apply to the robust performance in dealing with noisy incomplete cancer incidence data at low doses to disclose the threshold or the dose-proportionate increase in cancer risk. When analyzing the LSS cancer databases of the RERF, the method unequivocally disclosed the threshold for some cancers where survivors were most likely exposed to an uppermost organ dose of 0.1–0.2 Sv. Surprisingly, however, instead of a zero effect or Gaussian white noise, the threshold appeared as a negative excess relative risk, indicating the suppression of spontaneously or environmentally arising background cancer.

## APPLICATION OF ANN THEOREM TO CANCER RISK STATISTICS

In a single perceptron model of ANN [13], the ‘integrate-and-fire’ response, or a special case of noise cancellation process, is described by

(1) $$S(x) = \varphi \left( {\sum\limits_{i = 1}^n {w_i f\,(x_i ) - \mu } } \right),$$

where φ(..) is a gain function, in which *f*(*x*_i_) is the *i*th input variable with weight *w*_i_, and *μ* is the threshold. When the weighted sum of variables exceeds the threshold, the system is activated. In the application of the ANN theorem to cancer risk assessment, the relative risk (*RR*) of cancer at radiation dose *x*, i.e. *f*(*x*), is regarded as an input variable, and we tentatively dropped the threshold term because the threshold in cancer development is not known *a priori*. Consequently, we will hereafter consider a simplified ANN for a model-free random pattern describing a weighted sum of *RR*:

(2) $$S_{RR} = \sum\limits_{i = 1}^n {w_i \, f\,(x_i ),} $$

where *n* is the number of datapoints and *w* is a weighting defined by humming distance *w*_i_ = *x*_i_ − *x*_i__−__1_. *RR* is denoted as the number of cancer cases in the exposed population relative to that in the control population. If the study population is sufficiently large and cancer is refractory to radiation doses, the *RR* may be randomly distributed around the mean *μ*_0_ = 1 (Gaussian white noise). Since the excess relative risk (*ERR*) is given by *ERR* = *RR* − 1, the weighted sum of *ERR* is expressed by

(3) $${S_{ERR}=\sum\limits_{i=1}^n {w_i \{ f\,(x_i ) - 1\} =\sum\limits_{i=1}^n {w_i f\,(x_i ) - \sum\limits_{i=1}^n {w_i } (1),}} {{\qquad or\quad S_{ERR}=\ } S_{RR} - x}}. $$

*S*_*RR*_ is optimized by fitting to a continuously differentiable polynomial function by the maximum likelihood method combined with bootstrap resampling with weighting by the inverse function of *RR*. The optimized *S*_*RR*_ and its differential at dose *x*, *RR*(*x*) and *ERR*(*x*), are expressed by continuous probability density functions:

(4) $$S_{RR} = \sum\limits_{i = 0}^m {\theta _i x^i ,\,\,\,\,\,\,\,\,\,RR(x)} = \displaystyle{d \over {dx}}S_{RR} = \sum\limits_{i = 1}^m {i \times \theta _i \times x^{i - 1} } ,\,\,\,\,\,\,\,\,\,ERR(x) = RR(x) - 1,$$

where *θ*_*i*_ is the coefficient of the *i*th term of the polynomial. The optimal degree of polynomial, *m*, was determined by Akaike information criterion (AIC) [14] that minimizes

(5) $$AIC(m) = n\left\{ {1 + \ln (2\pi \,s^2 )} \right\} + 2(m + 1),$$

where *s*^2^ is the maximum likelihood.

## DATA SOURCES AND DATA ASSESSMENT FOR ANN ANALYSIS

The ANN statistics test was performed on two cancer databases, ‘lssinc07’ and ‘DS02can’, of the LSS cohort of A-bomb survivors, which are publicly available from RERF (http://www.rerf.or.jp). The database ‘lssinc07’ is the source term data used in Preston *et al.* [1] and contains cancer incidence (morbidity) data for 111 952 subjects followed up during the period 1958–1998, in which information on the occurrence of cancer (*X*) and its site is cross-tabulated in a total of 26 806 data cells (records) with city (*c*), gender (*s*), person year at risk (*PY*), age at the time of bombing (ATB, *a*), attained age (*t*), radiation dose (*x*) determined by the dosimetry system 2002 (DS02), and so on. The database ‘DS02can’ is the source term data used in Richardson *et al.* [2] and contains data on deaths (mortality) from all solid cancers combined or liquid cancers followed up during the 1950–2000 period on a total of 86 611 subjects compiled in 33 219 data cells, together with additional information as in ‘lssinc07’. Liquid cancers include leukemia, lymphoma and multiple myeloma, in which leukemia constitutes 46.5% of all cases. The analyses were carried out for solid cancer in the ‘lssinc07’ incidence database and for liquid cancer in the ‘DS02can’ mortality database. Subjects exposed to doses below 0.005 Gy, including persons who were not in the city at the time of bombing (NIC), were treated as controls. The *RR* was calculated according to the formula *RR* = [*X*/*PY*]_csat_/λ_csat_, where *X* is the observed number of cancer cases in the exposed population, and λ_csat_ is the expected frequency in the control population, in which cancer rate is expressed as a logistic function of attained age (*t*), λ_csat_ = *u*/[1 + *k*_1_·exp(−*k*_2_*t*)], for each city-, gender- and ATB-category. The calculation of age-dependency, *t* and *a*, was made with a 5-year interval. Neutron dose was weighted by the dose-dependent variable relative biological effectiveness (RBE) against γ-rays, *R*_γ,n_, as previously described [15], except for dose to the skin in which only neutron dose weighted by constant RBE = 10 was available. Liver dose was applied to organs in the upper trunk, i.e. oral, brain, esophagus, liver, breast, lung, stomach, gallbladder, pancreatic and thyroid cancers, and the colon dose was applied to all solid cancers combined and organs in the lower trunk, i.e. colon, rectum, ovary, cervix, urinary organ and prostate cancers. Bone marrow dose was used for liquid cancer.

To avoid systematic distortion of the distribution of *RR* around the mean, a population size that was large enough to satisfy *RR* > 0 was determined by moving window averaging (MWA). For instance, an MWA = w1000s50 implies that a group of 1000 data cells is consecutively moved forward in increasing order of dose with a step size of 50 data cells. The mean dose of the window is expressed as the *PY*-weighted dose. Although the minimum window size that satisfied *RR* > 0 was w100 for most solid cancers and w500 for liquid and thyroid cancers, the analysis was carried out with an MWA = w1500s50 and a cut-off dose to survivors, *D*_max_, of 3 Sv unless otherwise stated.

## THRESHOLD AT LOW DOSES IS MANIFESTED AS A NEGATIVE *ERR*

To begin, we performed a cross-validation of methods—the conventional piecewise dose-category method (e.g*.* [1]) and the present ANN method—used to calculate the *ERR* of solid cancers in a combined Hiroshima and Nagasaki cohort. To avoid any effects of the use of different neutron weighting systems, the analysis was performed using a constant RBE = 10 for neutrons. The results of the two methods matched reasonably well for the overall pattern of dose–response; including an abnormally elevated *ERR* at low doses (see Supplementary figure Fig. S1). However, unless otherwise stated, the ANN analysis was performed using the dose-dependent variable RBE of neutrons (RBE = *R*_γ,n_), and extended to the characterization of city differences, gender specificity and ATB effects with particular attention paid to the response at low doses.

**Fig. 1.**
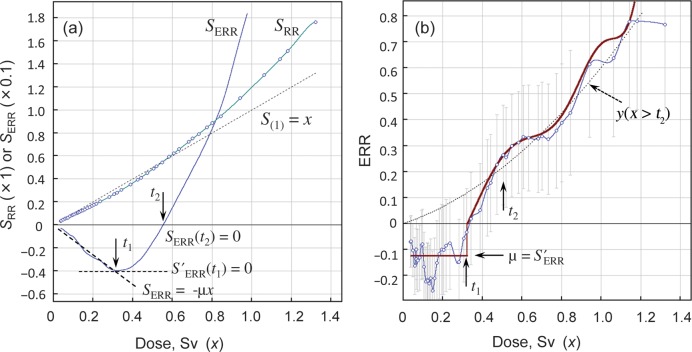
Data processing by the ANN method (example). Lung cancer incidence in Hiroshima A-bomb male survivors exposed at ATB (20+). MWA = w1000s50, D_max_= 3 Sv. **(a)** *S*_*RR*_ and *S*_*ERR*_ are the weighted sum of *RR* and *ERR*, respectively, fitted to a polynomial function of dose by maximum likelihood method. **(b)** The continuous probability density function of *ERR* (solid line) obtained by calculating *S*′_*RR*_ − 1 according to Eq. 4 is compared with the observed *ERR* data with 80% confidence intervals (CIs) connected by spline interpolation. *t*_1_ = 0.32, *t*_2_ = 0.55, *μ* = −0.12. The reference dose–response (dotted line) is calculated according to: *y*(*x* > *t*_2_) = (0.359 ± 0.066)*x* + (0.219 ± 0.066)*x*^2^.

The procedures of the ANN analysis are shown in Fig. 1 using an example of lung cancer cases in Hiroshima male survivors exposed at ATB (20+), this being 20 years or older at the time of bombing. Unexpectedly, the integrated *ERR*, *S*_ERR_ = *S*_*RR*__−_ *x*, was less than zero at low doses, it progressively decreased with dose to reach a minimum, and then increased with further increases in dose (Fig. 1a). This implies that, instead of zero risk (canonical definition of the threshold or random Gaussian fluctuation), the threshold was identified as a negative *ERR*, or manifested as a reduction of the background cancer rate, in a dose-independent manner at low doses. Here we call this negative *ERR* at low doses the ‘threshold’ although it differs from the conventional definition. The dose limit of the threshold, *t*_1_, was determined that satisfies the derivative *S*′_ERR_ = 0 by the Lagrange derivative interpolation method. The linearly decreasing part (*x ≤ t*_1_) of *S*_ERR_ was fitted to a linear regression *S*_ERR_ = κ+ *μx*, the derivative of which gives the *ERR* at threshold, *ERR* = *μ*. The dose, *t*_2_, at which *S*_ERR_ crosses the *S*_ERR_ = 0 axis, corresponds to the dose above which all survivors within a moving block were exposed to a supra-threshold dose of radiation (dose *x* > *t*_1_). Thus, the *ERR* is discontinuous with a breakpoint at *t*_1_, followed by a transient phase *t*_1_ < *x* < *t*_2_, and then a dose range without threshold response (*x* > *t*_2_; Fig. 1b). The suppression-independent, non-threshold, dose–response may be constructed only for *ERR* datapoints in the dose range *x* > *t*_2_ (dotted line in Fig. 1b). We call this the ‘reference dose–response’, which represents induction kinetics unrelated to the low-dose suppression, or the universally valid induction function; this can be fitted to a linear-quadratic function of dose described by the equation: *y*(*x > t*_*2*_) = *αx* + *βx*^2^. Parameters for the threshold and polynomial coefficients of the best fit to the probability density function of *ERR* calculated in this way for each cancer type are presented in Supplementary data Table S1.

## SOLID CANCERS: RESPONSE CHARACTERISTICS BY CITY, GENDER AND CANCER SITE

**Fig. 2.**
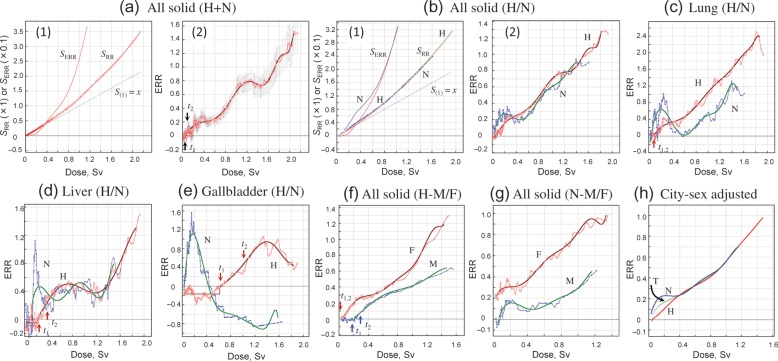
Solid cancers by city and gender. **(a)** All solid cancers in a combined Hiroshima and Nagasaki cohort (T). Panel 1: *S*_*RR*_ and *S*_ERR_. Panel 2: *ERR*. **(b)** City-based difference of all solid cancers. **(c)** Difference by city for *ERR* for lung cancer. **(d)** Difference by city for *ERR* for liver cancer. **(e)** Difference by city for *ERR* for gallbladder cancer. **(f)** Gender difference for *ERR* for all solid cancers in Hiroshima. **(g)** Gender difference for *ERR* for all solid cancers in Nagasaki. **(h)** City- and gender-adjusted *ERR* for all solid cancers in general populations with anticipated 1:1 gender ratio. H = Hiroshima, N = Nagasaki, T = two cities combined, M = male. F = female.

Figure 2a shows the ANN analysis of all solid cancers in a combined Hiroshima and Nagasaki cohort. Although the neutron dose was weighted by dose-dependent RBE, an overall dose–response was not much different from that obtained using an RBE = 10. A small-scale threshold was indicative in *S*_ERR_ response at low doses (Fig. 2a-1), which was followed by an atypical elevation of *ERR* at 0.2–0.4 Sv (Fig. 2a-2). However, when the data for Hiroshima and Nagasaki survivors were treated separately, it became evident that this elevation of *ERR* at low doses was a reflection of the situation in Nagasaki survivors (Fig. 2b), and mainly due to cancers of the lung, liver and gallbladder (Fig. 2c–e), but not to other solid cancers included in the screening protocol. When males and females were treated separately, the threshold was evident in males in Hiroshima (Fig. 2f). However, in Nagasaki, a transient elevation of *ERR* at low doses occurred both in males and females and tended to mask the threshold that was potentially present in survivors from that city (Fig. 2g).

A significant difference in dose–response pattern between the two cities of Hiroshima and Nagasaki at low doses may give rise to a problem in the use of combined two-city data for risk projection extrapolated to the general population. The male/female ratio differed considerably from unity, as was also the case for other areas during the second world war, and varied with ATB; for instance, it was 0.3–0.4 for ATB (20–40), whereas it was 0.8–0.9 for ATB (0–20) and ATB (40–60). Since the same trend was also the case for control populations, such age structures might have had little effect on the relative risk of cancer for each sex. Adjustments for the city in question may however be needed because, in terms of population size, the Hiroshima-to-Nagasaki ratio of the exposed populations is 0.76:0.24. Figure 2h shows the *ERR* of solid cancers in an anticipated 1:1 gender ratio for Hiroshima (H), Nagasaki (N) and combined-city (T) populations after adjustment for city ratio and gender ratio differences. As expected, adjustment according to the gender ratio distribution among ATB groups had no effect on outcome. In contrast, however, although the Nagasaki cohort comprised 24% of the total combined population, an abnormal elevation of *ERR* at low doses (≤0.4 Sv) was still apparent for the two cities combined, and hindered the construction of a joint dose–response relationship in a simple form. The reference dose–responses (threshold- and low-dose abnormality-unrelated) are described by *y*_H(__*x*>*t*__2)_ = (0.501 ± 0.065)*x* + (0.088 ± 0.008)*x*^2^, *y*_N(__*x*>0.4)_ = (0.512 ± 0.017)*x* + (0.061 ± 0.020)*x*^2^ and *y*_T(__*x* > 0.4)_ = (0.511 ± 0.070)*x* + (0.057 ± 0.081)*x*^2^, for Hiroshima (H), Nagasaki (N) and the two cities combined (T), respectively. The reference dose–responses were very close to each other at high doses, and characterized by the presence of a small but significant dose-quadratic term.

**Fig. 3.**
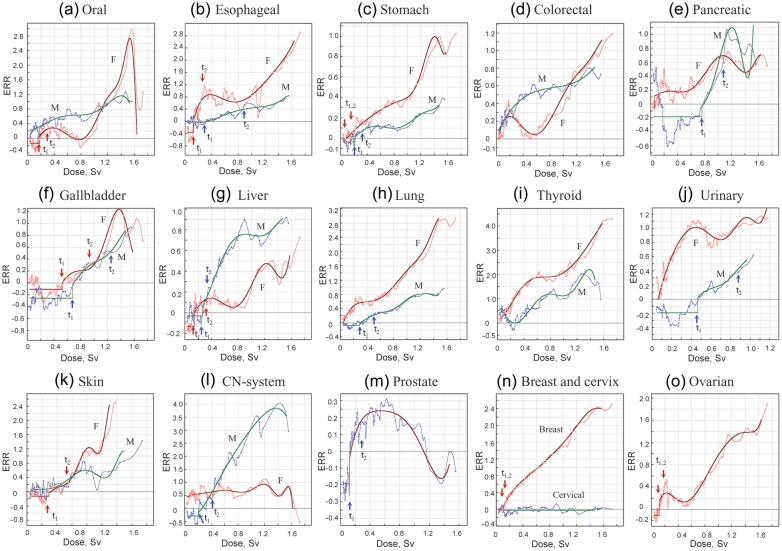
Difference in *ERR* by gender and organ (Hiroshima). **(a)** Oral cancer. **(b)** Esophageal cancer. **(c)** Stomach cancer. **(d)** Colorectal cancer. **(e)** Pancreatic cancer. **(f)** Gallbladder cancer. **(g)** Liver cancer. **(h)** Lung cancer. **(i)** Thyroid cancer. **(j)** Cancer of urinary system. **(k)** Skin cancer (non-melanoma). **(l)** Brain cancer (cancer of central nervous system). **(m)** Prostate cancer. **(n)** Breast and cervical cancer. **(o)** Ovarian cancer. M = male, F = female.

The ANN statistics were then applied for to analyze organ specificity and gender specificity in Hiroshima survivors (Fig. 3). The threshold level differed by cancer site and gender, being prominent in males for cancers of the esophagus, stomach, pancreas, gallbladder, liver, lung, urinary organs and brain (central nervous system), and both in males and females for skin cancer. The threshold was also seen in breast, prostate and ovarian cancer, but not in colorectal or thyroid cancer for males or for females. A small threshold was evidenced for oral cancer in females but not in males. Breast cancer in females increased proportionally to dose following a small threshold at low doses (*t*_1_ = 0.107 Sv, *μ* = − 0.022). This contrasted to cervical cancer, which showed a complete lack of response to dosage (Fig. 3n). Such was also the case for breast and cervical cancer in Nagasaki (data not shown), indicating a prevailing role of human papillomavirus (HPV) in the causation of cervical cancer.

## LIQUID CANCERS, INCLUDING LEUKEMIA

**Fig. 4.**
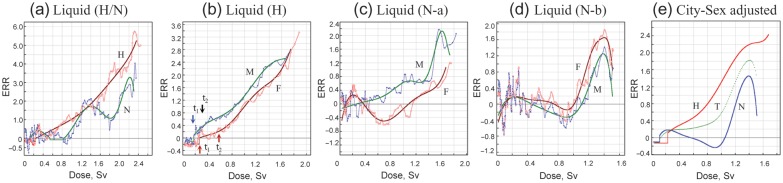
Liquid cancer. **(a)** City differences for *ERR*. **(b)** Gender difference for *ERR* in Hiroshima. **(c)** Gender difference for *ERR* in Nagasaki (including factory workers) (N − a). **(d)** Gender difference for *ERR* in Nagasaki (excluding factory workers) (N − b). **(e)** The dose–responses after city- and gender-adjustment. For Nagasaki, adjustments were made using non-factory workers. M = male, F = female, H = Hiroshima, N = Nagasaki, T = two cities combined.

Liquid cancers (combined leukemia, lymphoma and multiple myeloma) were studied in the mortality data. The dose–response of *ERR* was quite different between cities (Fig. 4a). In Hiroshima, the dose–response of *ERR* was similar to that of incidence (morbidity) data for solid cancers described above; i.e. there was a distinct threshold that was followed by a dose-proportional increase in *ERR* both in males and females (Fig. 4b). The reference dose–response was y_(__*x* > t2)_ = (0.924 ± 0.066)*x* + (0.563 ± 0.053)*x*^2^ and y_(__*x* > t2)_ = (0.888 ± 0.147)*x* + (0.737 ± 0.104)*x*^2^, for males and females, respectively. The threshold parameters were *t*_1_ = 0.187 Sv, *t*_2_ = 0.271 and *μ* = − 0.105 for males and *t*_1_ = 0.239 Sv, *t*_2_ = 0.598 and *μ* = − 0.190 for females. For the Nagasaki data, the dose–response patterns differed not only from those of Hiroshima but also by gender (Fig. 4c). Surprisingly, however, when factory workers were excluded from the analysis, gender differences completely disappeared (Fig. 4d). However, the overall response pattern still differed from that of Hiroshima in that it showed a large-scale threshold expanding over a wide dose range up to 1 Sv, although it was superimposed by a transient increase of risk at low doses as seen for solid cancers in Nagasaki. Because of this unique feature of Nagasaki survivors, data for the two cities cannot be combined for generalization. Figure 4e shows the gender-adjusted dose–response for Hiroshima (H), Nagasaki (N, non-factory workers) and the two cities combined (T) cohorts. In Hiroshima, the *ERR* increases in a relatively monotonic fashion following the appearance of a distinct threshold at low doses. This contrasts to Nagasaki, where a small increase in *ERR* at low doses was followed by a long-range suppression of *ERR* up to 1 Sv, and then by a dose-proportional increase at higher doses. The increase at high doses was comparable to that in Hiroshima. For generality, the reference dose–response could be represented only by that of Hiroshima, which is *y*_H(__*x* > *t*__2)_ = (0.433 ± 0.025)*x* + (0.731 ± 0.028)*x*^2^, where *t*_1_ = 0.178 Sv, *t*_2_ = 0.404 Sv and *μ* = − 0.125. The response kinetics was very similar to those of solid cancers.

## EFFECTS OF AGE AT THE TIME OF BOMBING (ATB)

**Fig. 5.**
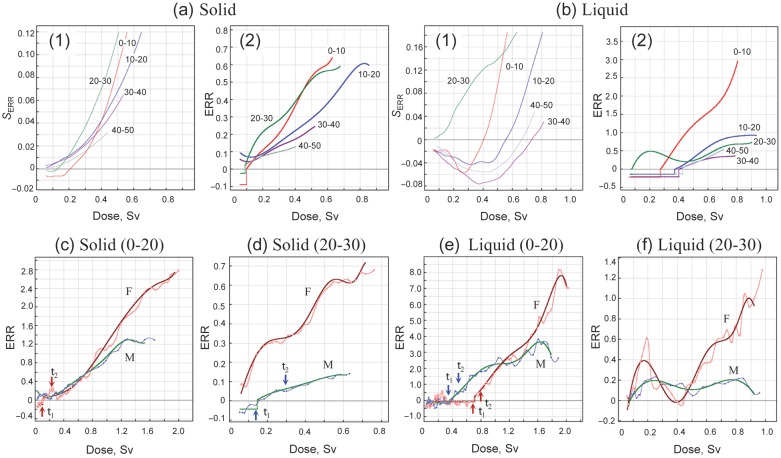
Effects of ATB (Hiroshima). **(a)** Solid cancers. **(b)** Liquid cancers. Panel 1 shows *S*_ERR_ and Panel 2 shows *ERR* at each ATB. **(c)** *ERR* for solid cancers for ATB (0–20). **(d)** *ERR* for solid cancers for ATB (20–30). **(e)** *ERR* for liquid cancers for ATB (0–20). **(f)** *ERR* for liquid cancers for ATB (20–30). M = male, F = female.

The effects of age at the time of bombing (ATB effects) were studied in Hiroshima survivors, with the results summarized in Fig. 5. The threshold was more pronounced in younger ATB groups for solid cancers, being particularly more evident for liquid cancers (Fig. 5a and b). This can be seen more clearly in the weighted sum of *ERR* (Fig. 5a-1 and b-1). The age-dependence of *ERR* was generally the same as that reported previously [16–18], in that the *ERR* was higher for younger ATB both for solid and liquid cancers (Fig. 5a and b). An exception to this rule was ATB (20–30), which showed the highest *ERR* at low doses. Since the male-to-female ratio of this group was low (0.29:1), the *ERR* patterns for males and females were treated in proportion to the gender ratio and compared with those of ATB (0–20), where the male-to-female ratio was nearly 1:1. As seen in Fig. 5c–f, the atypical aspect of the ATB (20–30) group was due to the elevated *ERR* at low doses in female survivors, both for solid and liquid cancers. The reason for this is not clear, but may be due to an artificial downwards shift of the dose rather than a consequence of biological factors. In the early years after the bombing, when knowledge of the heritable effects was lacking, it is likely that there were some biases in registering dose-related exposure conditions experienced by young unmarried women. Indeed, a decreased marriage rate was noted for Hiroshima and Nagasaki women of young ATB due probably to the anticipated discriminatory bias for marriage [19].

## DISCUSSION AND CONCLUSION

**Fig. 6.**
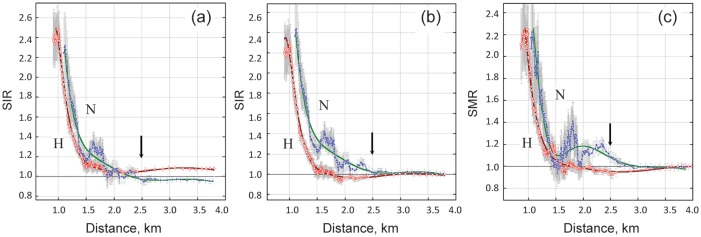
Distribution of *SIR* and *SMR* of solid cancers with ground distance from the hypocenter. **(a)** *SIR* standardized by NIC as the reference population (not normalized). **(b)** *SIR* standardized by NIC as the reference population (normalized by farthest value set at *SIR* = 1). **(c)** *SMR* standardized by nominal control population (<0.005 Gy) as the reference population (normalized by farthest value being set at *SMR* = 1). Arrows indicate the critical distance beyond which no survivors were exposed to doses of *x* ≥ 0.005 Gy.

A new approach to using ANN statistics has been shown here to provide a powerful means for defining cancer risk in response to exposure to low doses of radiation. The systematic distortion of the dose–response pattern of cancer risk at low doses of radiation exposure was evident in relation to (i) an abnormal elevation of risk for some cancers, specifically in Nagasaki, and (ii) the organ- and gender-specific appearance of a threshold, appearing as a negative excess relative risk at low doses. The origin of the transient heightening of cancer risk at low doses of radiation exposure in Nagasaki remains to be elucidated, but it nevertheless had an impact on the overall dose–response pattern of cancer risk. For any effects of radiation to be observed other than direct flash radiation, such as the spreading of radioactive fallout and/or secondary radionuclides, the distribution of standardized incidence (or mortality) ratio, *SIR* or *SMR*, of solid cancers against ground distance from the hypocenter must be studied. *SIR* and *SMR* were calculated using the ANN method, as described above for *RR*, i.e. *SIR* = *RR* = [*X*/*PY*]_csat_/λ_csat_, but in this case as a function of ground distance from the hypocenter rather than radiation dose (Fig. 6). Cancer incidence was standardized in the NIC cohort that constituted residents who were not in the city at the time of the bombing. In terms of ground distance from the hypocenter, the controls (<0.005 Gy) correspond to those at about 2.5 km or farther from the hypocenter. In Hiroshima, the *SIR* in distal survivors (<0.005 Gy) was significantly higher than unity (Fig. 6a). A similar result was noted by Watanabe *et al.* [20], who used the entire populations of the Hiroshima and Okayama prefectures as reference populations. A monotonic increase in *SIR* in distal survivors over a wide range of ground distance suggests that this elevated level of *SIR* could be a false positive result due to geographic variability in the reference populations, as suggested by Grant *et al.* [21]. The *SIR* was then normalized to that of the farthest distance, which was 3.84 km (range, 2.30–7.58 km) for Hiroshima and 3.79 km (2.49–6.97 km) for Nagasaki. As seen in Fig. 6b, there was no indication of an increase in *SIR* that could not be accounted for by primary flash radiations in Hiroshima. However, in Nagasaki, the elevation of *SIR* continued by passing across the 2.5 km point and beyond. The same trend was also observed for *SMR* (Fig. 6c), in which the mortality ratio was standardized in the control cohort because the ‘DS02can’ mortality database did not include NIC groups.

The long tail of the *SIR* or *SMR* in Nagasaki is likely to be a reflection of continued exposure to alpha particles emitted from internally deposited ^239^Pu. Indeed, ^239^Pu fallout has been observed in the eastern area of Nagasaki at a distance ≥ 1 km from the hypocenter [22–25]. Furthermore, an unusually high level of alpha tracks has been observed in autopsy samples from Nagasaki victims [26]. The biological half-life of ^239^Pu (fuel material in the Nagasaki bomb) is about 200 years, which is extremely long compared with the 15–100 days for the ^235^U used in the Hiroshima bomb [27].

A most interesting finding made here was the threshold response at low doses, which was unique in that it manifested as a negative *ERR*, or in other words a suppression of the background cancer rate. The biological mechanism behind this threshold response is unclear. However, allowing for a considerable variability in the estimated dose within an organ, the critical dose below 0.1–0.2 Sv is reminiscent of that for the radio-adaptive response (ADR) [28–30]. ADR is a well-documented phenomenon by which irradiation with low linear energy transfer (LET) radiation, such as X- or γ-rays, at low doses (typically < 0.1 Gy) renders cells resistant to the induction of mutation and chromosome aberrations by subsequent exposures to radiation or chemical genotoxins. The low doses of low-LET radiation alone reduce spontaneously occurring chromosome aberrations and malignant transformation in cultured mammalian cells [31–34]. Moreover, irradiation of mice with low dose, or low dose-rate, γ-rays has been reported to suppress spontaneously occurring as well as chemical-induced mutations and tumors [35–42]. All available evidence points the possibility that low-doses of low-LET radiation activate a sustained-response, error-free DNA repair system.

Obviously, the challenge of understanding the underlying molecular basis of cancer epidemiology requires further research and discussion. It is tempting nevertheless to speculate on possibilities within the context of recently emerging experimental evidence concerning the DNA repair pathway choice of cells in response to DNA double-strand breaks (DSBs). DSBs are produced by ionizing radiation, DNA replication forks stalled by bulky adducts, or by other causes, and present a major threat to genetic integrity; they are, consequently, a leading cause of chromosome aberrations and cancer in cells exposed to ionizing radiation and chemicals [43, 44]. DSBs are repaired either by homologous recombination (HR) or by non-homologous end-joining (NHEJ) processes that are present in cells as distinct and competitively operating pathways [45, 46]. Recently, two subpathways have been identified for NHEJ, i.e. canonical NHEJ (C-NHEJ, also called D-NHEJ or the DNA-PK-dependent pathway) and alternative NHEJ (Alt-NHEJ, also known as B-NHEJ or the backup pathway) [refs. 47–49 for review]. The molecular nature and biological significance of these pathways are distinct; C-NHEJ is a rapid and precise high-fidelity process that mediates direct joining of two broken ends, whereas Alt-NHEJ is a slow and error-prone process thought to be a primary mediator of mutations since it often requires end processing and uses sequence homology of various lengths at a junction (microhomology-mediated end-joining, MMEJ) [50]. Briefly, the former employs Ku70/80 end-binding proteins associated with DNA-PKcs to form a holoenzyme that phosphorylates Artemis (*Cernunnos*) endonuclease, DNA polymerase μ/λ, and the XRCC4/ligase IV/XLF complex for ligation, whereas the latter uses PARP1 for DSB recognition followed by CtIP/MRN endonuclease, DNA polymerase β and XRCC1/ligase III. In mammalian cells, the two NHEJ pathways have been proposed to contribute equally [51]. HR is initiated by DSBs generated during replication, where the DSB end is resected to give rise to a 3′ overhang, which recruits Rad51 recombinase and invades an intact homologous duplex to use it as a template for repair by polymerase δ/ε and ligase I. HR is often described as ‘error-free’, but its recombination processes at replication arrest can result in large-scale genome rearrangements such as deletions and translocations that could contribute to cancer [52–54]. Low doses of low-LET radiation stimulate the DNA-PKcs/Ku-dependent NHEJ pathway, and then render cells resistant to subsequent high doses of radiation [55]. The choice of the precise rejoining pathway by low-dose irradiation has been confirmed in the *in vitro* DSB rejoining assay system [56, 57], where the high fidelity rejoining pathway is activated in low-dose irradiated cells, which in turn suppresses microhomology-mediated misrejoining that is otherwise observed in high-dose irradiated cells. Similar to the dose limit for ADR [29] the critical dose for pathway switching is ∼ 0.1 Gy [58].

A most significant observation of the repair process is the crosstalk among repair pathways; i.e. the activation of C-NHEJ suppresses Alt-NHEJ and HR [47–49]. The discriminating signaling processes, e.g. cell cycle phase dependence, end-stabilization (Ku70/80 vs PARP1), end-processing (inhibition by Rif1/53BP) and/or kinase specificity (DNA-PKcs vs ATM/ATR), have been reported for their co-regulation and pathway choice [59–77]. It is noteworthy that 53BP1 (p53 binding protein) is essential for the Ku70/DNA-PKcs/Artemis-dependent NHEJ pathway (C-NHEJ), but not for HR [74, 75]. Rif1 (Rap1 interaction factor 1) cooperates with 53BP1 to inhibit the 5' end resection needed for Alt-NHEJ and HR [76, 77]. Interaction between tumor suppressor protein p53 and 53BP1 is crucial for end resection, where 53BP1 binds to the core residue at the BRCT domain but not to mutant p53 [78]. These lines of evidence are consistent with cytogenetic observations that activation of the ADR response is strictly dependent on wild-type p53 but refractory to mutant p53 or abrogation of p53 by transcriptional silencing [29, 30, 79, 80]. The p53 is directly phosphorylated by p38MAPK [81, 82]. We previously found that p38MAPK is specifically activated by low-dose X-rays but downregulated by high doses [83]. p38MAPK has been also reported to mediate dose-dependent biphasic activation of signaling molecules by γ-rays and hydrogen peroxide [84–86]. It is thus likely that the p38MAPK/p53/53BP1 damage-sensing network plays a pivotal role in ensuring a switching DSB repair pathway to error-free mode. During S phase, BRCA1 antagonizes 53BP1, where there is also redundancy with p53 [77, 87–89]. The impairment of HR has been described as suppressing spontaneous and radiation- or chemically induced sister-chromatid exchange (SCE, a cytogenetic manifestation of HR) [90] and chromatid aberration, but at a cost of cell killing [91–94]. In support of this, low-dose irradiation renders cells hypersensitive to killing [95] and reduces chemically induced SCE [96].

Unlike the direct breakage of the DNA duplex by ionizing radiation, DSBs are also generated when replicating DNA encounters endogenous lesions (mostly single-strand breaks) or lesions associated with exogenous genotoxins including ultraviolet light (UV) and chemicals. The replicating DNA disrupted at the replication fork by DNA lesions may eventually be collapsed, producing DSBs that are subject to repair by HR and NHEJ. The suppression of error-prone HR and Alt-NHEJ by low-dose radiation may reduce chromosome aberrations and erroneous crossover (albeit enhancing cell killing) and hence mitigate cancer. Recently, DSBs of endogenous origin have been estimated to be 20–100 times lower in prevalence than previously estimated, and more likely to occur at a rate of ∼ 1 DSB/replication cycle in the human genome [97]. This raises the importance of DNA lesions associated with exogenous genotoxins, such as metabolites of cigarette smoke, alcohol, environmental sources, nutrition and dietary mutagens, which are major causes of human cancer [98–100]. The prevalence of threshold in cancer of the esophagus, lung, stomach and pancreas in male survivors suggests that smoking and alcohol consumption could be significant factors, since they are major cancers causally related to smoking and alcohol consumption [101]. Tobacco smoke contains at least 60 chemical carcinogens, of which many form bulky DNA adducts [102]. Acetaldehyde, a metabolite of ethanol, also forms DNA adducts and is causally related to the development of various cancers of the gastrointestinal tract, typically esophageal cancer [103]. The DNA adducts are removed by base excision repair (BER) or nucleotide excision repair (NER), or when they meet to DNA replication stall replication at fork and give rise to DSB, which is subject to repair by HR [104]. UV-induced lesions also cause replication arrest, leading to the development of DSBs and activation of HR; these processes have been correlated with the development of skin cancer [105]. Collectively, it is likely that the HR pathway is repressed by low-dose ionizing radiation, reducing the mutagenic rejoining at fork arrests and eventually suppressing cancer associated with smoking, alcohol and UV. Given the interplay between low-dose radiation and genotoxins, of serious concern is the fact that the acetaldehyde-detoxifying gene, aldehyde dehydrogenase-2 (*ALDH2*) is polymorphic, and its inactive variant ALDH2*Glu540Lys is prevalent in east Asian populations (about 40% in Japan) in contrast to populations of Caucasian ancestry where the prevalence of this variant is almost nil [106, 107].

Smoking and alcohol consumption are also risk factors for the development of oral cancer. While the dose–response pattern of *ERR* was similar to that of esophageal cancer (Fig. 3a and b), the low-dose threshold was not observed in males. The reason for this is not clear, but it could be related to the interaction of two risk factors that are supermultiplicative for oral cancer and submultiplicative for esophageal cancer [108]. Moreover, an involvement of human papilloma viruses has also been noted for oral cancer [109].

The reason for the unique dose–response pattern for liquid cancers in Nagasaki remains an open question. Difference associated with gender, and their disappearance when factory workers were excluded from the cohort analysis, strongly suggest an involvement of adult T-cell leukemia/lymphoma (ATLL), because the ATLL is endemic in southwestern Japan, including Nagasaki, and the factory workers were not necessarily Nagasaki native citizens, but many would have been conscripted or mobilized from other parts of Japan. ATLL is a pathological manifestation of HTLV-1 infection with long-term latency period. The infection itself is asymptomatic but the stable integration of viral DNA into transcriptionally active regions of the host genome is critical for the onset of disease [110]. The integration of retroviral DNA is a multistep process and anticipated to be associated with the DSB repair system [111]. The role played by DSB repair mechanisms in retroviral integration remains unclear and is not free of controversy; some cell-based studies have shown that deficiencies in early sensors of NHEJ, i.e. DNA-PK kinase and MRN nuclease, or overexpression of Rad51 or Rad52 recombination proteins, but not other Rad52 paralogs, downregulate the retroviral integration [112–117], while others have shown that damage sensors for NHEJ, including DNA-PK, are not critical [118]. The suppressive effects of Rad51/Rad52 overexpression could be due to a steric hindrance of integrase, a retroviral recombinase, by competition between functional domains shared by the two proteins [113, 119], since the overexpression of Rad51 is known to suppress DSB-induced HR [120, 121]. For instance, in cells infected with HIV-1 (human immunodeficiency virus 1), Rad51 proteins (a major component of HR) are recruited and promote viral DNA integration [122]. On the other hand, the activation of DNA-PK, a key component of C-NHEJ, inhibits retroviral DNA integration [123]. These findings are consistent with the DSB repair pathway choice in response to low-dose radiation; i.e. suppression of HR by activation of the C-NHEJ pathway. Since the impairment of HR does not alter the efficiency of NHEJ [124], HR may play a critical role in the retroviral integration. Therefore, the suppression of liquid cancers at low radiation doses (<1 Sv) in Nagasaki could be a consequence of the activation of the C-NHEJ pathway by low doses of radiation, leading to downregulation of HR-mediated HTLV-1 DNA integration in virus carriers. Incidentally, in a recent survey dealing with ATLL in Nagasaki, HTLV-1 seroprevalence and lifetime risk of ATLL in carriers were found to be 11.82 and 7.29, and 15.6 and 3.78%, for males and females, respectively [125]. The lifetime HTLV-1 prevalence rate in non-endemic areas of Japan is currently about 1.5% although a progressive yearly increase has been noted that is due to population movement [126]. The inclusion of migrant factory workers from non-endemic areas may have distorted the dose–response of liquid cancers, particularly at low radiation doses.

Here, the low-dose response of cancer induction has been discussed in favor of the DSB repair pathway choice. For the pathway choice to have an effect, the activation should be sustainable for a long period of time after exposure. ADR by low doses of low LET radiation has been associated with the enhanced repair of DSBs by C-NHEJ pathway, since it is dependent on Ku and DNA-PKcs [127, 128] and inhibited by high-LET radiation [129]. The experimental evidence for the sustainability of ADR is not consistent; it rarely exceeds 2 d in mammalian cells cultured *in vitro*. However, it is much longer in animals irradiated *in vivo*, e.g. lasting at least for weeks, months, and even life-long depending on the experimental protocol used [130–132]. Thus, the sustainability of the signaling process seems to be different between cultured cells and whole body, probably due to its incorporation in stem cells in the latter. ADR is also inducible in spermatogonial cells of mice, but it is not transmitted to offspring [131]. In A-bomb survivors, radiation exposure influences the risk of lung cancer associated with tobacco smoking, including post-bombing smoking history, where the joint effects are less than additive; in this way it has been reported that radiation exposure tends to decrease the risk of smoking-associated lung cancer more efficiently in heavier smokers [133–135]. A more pronounced effect of radiation exposure on heavier smokers is likely because the repair mechanism depends on the types and amount of damage in the genome. Frankenberg-Schwager *et al.* [136] showed that, in the repair of post-replication-derived DSBs, HR is more important than NHEJ for the repair of complex DSBs, while NHEJ plays a major role in the repair of simple DSBs. Unlike HR, status of C-NHEJ and p53 does not affect the efficiency of other pathways such as NER, mismatch repair (MMR) and SSB repair (TDP1) [80]. Tobacco smoking- and alcohol-related cancers are not simply proportional to the dose, but often show a J-shaped dose–response reflecting a different burden on the repair pathways depending on the amount of damage to the genome [137–139]. In this context, evidence is accumulating concerning the epigenetic memory of the DSB repair pathway choice, namely a crosstalk between the particular type of DSB repair and histone modification, called ‘histone code’ theory [140–142]. The tail domains of histones are modified by acetylation, methylation, phosphorylation or sumoylation in a manner that is specific to the repair pathway, i.e*.* NHEJ or HR [143, 144]. It is noteworthy that the lifetime epigenetic memory of the stress response in *Drosophila* is associated with the phosphorylation of activating transcription factor 2 (ATF2) by stress-activated protein kinase p38MAPK [145]. Indeed, ATF2 is activated in response to low doses of X-rays by p38MAPK via PKCα-p38MAPK-PLCδ autoregulatory circuitry signaling loops and downregulated by high doses [38, 146].

## PERSPECTIVES

Due largely to a limited statistical power at low doses in A-bomb survivors, cancer risk is often expediently correlated linearly with dose down to zero dose without threshold and expressed on a ‘per-Sv’ basis [147]. The application of the ANN method developed here circumvents this difficulty and unequivocally demonstrates for the first time the presence of a threshold of excess relative risk in humans exposed to ionizing radiation. However, the threshold was fundamentally different from that of the canonical definition of zero effect until the dose reached a critical point, but instead it was manifested as a reduction of background cancer rate. Therefore, cancer risk at low doses is not a simple extension of that seen with moderate-to-high radiation doses. Obviously, much remains to be determined when considering the reasons underlying such a unique response. Here we hypothesized that the response should be considered in the context of radiation–environment interplay, favoring recently emerging experimental evidence involving DSB repair pathway choice and epigenetic memory by histone code theory. In this way, activation of the high fidelity C-NHEJ (or ADR) pathway, although not excluding joining of illegitimate ends, by low doses of low-LET radiation suppresses the microhomology-mediated error-prone Alt-NHEJ or HR pathways, and hence mitigates the genomic insult caused by DSBs arising from radiation exposure or fork arrest by genotoxins. Such a choice is not the case for high doses (>0.1 Gy) or high-LET radiation, which not only are unable to elicit ADR but also invalidate the ADR response to low-LET radiation [148, 149]. The low dose abnormality in Nagasaki could be a case in question.

Considering the appearance of the threshold as a consequence of radiation–environment interplay or lifetime genotoxin experience that is not intrinsic to radiation *per se*, the setting of radiation protection standards cannot be made on the premise of lifestyle due to vast individual variability. Otherwise, cancer risk will be underestimated for persons with a healthy lifestyle, children and for persons exposed to high-LET radiation. As a precautionary principle, we propose the use of a ‘reference dose–response’ function as a risk factor for a universally valid protection standard, which provides a threshold-unrelated measure of cancer risk (*x* > *t*_2_). In this way, a gender-averaged *ERR* is best expressed in the following ways:

$${\eqalign{& {\rm Solid \,cancers}\!:{\rm }y = (0.{\rm 5}0{\rm 1} \pm 0.0{\rm 65})x + \left( {0.0{\rm 88} \pm 0.00{\rm 8}} \right)x^{\rm 2} ,{\rm and} \cr & {\rm Liquid \, cancers}\!:y = \left( {0.{\rm 433} \pm 0.0{\rm 25}} \right)x + \left( {0.{\rm 731} \pm 0.0{\rm 28}} \right)x^{\rm 2} ,}}$$

where *x* is the radiation dose in Sv. With the exception of high-LET radiation, the response function itself does not necessarily provide a measure of risk assessment at low doses, because the extrapolation to zero dose, or the presence or absence of the threshold, is related to the unforeseen factors such as genotoxin experience, and probably of post-exposure history as well. The issue also evokes questions concerning the so-called ‘radiation hormesis’ (beneficial effect) and ‘healthy worker effect’ in some cancers in populations exposed to low doses of low-LET radiation [150–153]. These must be reconsidered in view of the negative interaction between radiation and environment. To date, confounding factors have often been considered in terms of additive or synergistic interactions. The present observations may lead to a new paradigm for explaining the molecular basis of cancer epidemiology in human populations exposed to low doses of ionizing radiation. Furthermore, granted the interplay between low doses of ionizing radiation and environmental carcinogens, caution must be taken in risk transferring across populations in the context of genetic polymorphisms of redox genes such as *ALDH2* and the prevalence of *HTLV-1*, in which disease mechanisms are closely related with the DSB repair pathway.

## SUPPLEMENTARY DATA

Supplementary data is available at the *Journal of Radiation Research* online.

## CONFLICT OF INTEREST

The authors have no conflicts of interest to report.

## Supplementary Material

Supplementary Data
