# Supplementary material for: Cancer risk at low doses of ionizing radiation: artificial neural networks inference from atomic bomb survivors
Source: J Radiat Res. 2013 Dec 22;55(3):391–406. doi: 10.1093/jrr/rrt133 (PMC4014156; doi:10.1093/jrr/rrt133)
Supplement: Supplementary Data [file supp_55_3_391__index.html]

Cancer risk at low doses of ionizing radiation: artificial neural networks inference from atomic bomb survivors — Supplementary Data 

# Cancer risk at low doses of ionizing radiation: artificial neural networks inference from atomic bomb survivors

## Supplementary Data

Supplementary Data

**Files in this Data Supplement:**

- Supplementary Data - Docx file
- Supplementary Figure - docx file
