# Supplementary material for: Cancer risk at low doses of ionizing radiation: artificial neural networks inference from atomic bomb survivors
Source: J Radiat Res. 2013 Dec 22;55(3):391–406. doi: 10.1093/jrr/rrt133 (PMC4014156; doi:10.1093/jrr/rrt133)
Supplement: Supplementary Data [file supp_rrt133_rrt133supp.docx]

**Supplementary data**

**Cancer risk at low doses of ionizing radiation: Artificial neural networks inference from atomic bomb survivors**

Masao S. SASAKI, Akira TACHIBANA and Shunichi TAKEDA

**[J. Radiat. Res., ***:***-***,****]**

**Table S1. Threshold parameters and regression coefficients of probability density function of excess relative risk (*ERR*)**.

The *ERR* is expressed by: *ERR*(*x*≤*t*_1_)=μ, *ERR*(*x*>*t*_1_)=*θ*_0_+*θ*_1_*x*+*θ*_2_*x*^2^+ . . . . . . . . . +*θ*_n_*x*^n^, (*x* is dose in Sv). Coefficient given by *θ*_k+3_=1.234-5 at *k*=10 should read *θ*_13_=1.234×10^-5^. H: Hiroshima, N: Nagasaki, *m*: male, *f*: female, *x*_u_: upper bound of dose.

(1) All solid cancers in Hiroshima (H) and Nagasaki (N) (Incidence)

| City and gender | *t*_1_ (Sv) | *t*_2_ (Sv) | μ | *x*_u(_(Sv) | *k* | *θ*_k+0_ | *θ*_k+1_ | *θ*_k+2_ | *θ*_k+3_ | *θ*_k+4_ | *θ*_k+5_ | *θ*_k+6_ | *θ*_k+7_ | *θ*_k+8_ | *θ*_k+9_ |
| --- | --- | --- | --- | --- | --- | --- | --- | --- | --- | --- | --- | --- | --- | --- | --- |
| Solid, (H+N) (*m*+*f*) | 0.034 | 0.084 | -0.033 | 2.14 | 0  10  20 | 1.201-2  -1.423-2  2.202-6 | 9.134-1  1.363-2  2.547-6 | 8.712-1  1.408-3  -4.875-6 | -1.104+0  3.542-3  1.277-7 | 2.317-1  -1.630-3  2.568-7 | 5.465-1  -9.351-4  3.370-8 | -1.981-2  -5.871-5  -4.154-8 | -1.798-1  1.515-4  -2.809-8 | -1.314-2  3.505-6 | 9.680-3  5.964-6 |
| Solid, H (*m*+*f*) | 0.044 | 0.082 | -0.018 | 1.93 | 0  10  20 | 2.650-3  4.828-2  6.965-5 | 8.813-1  1.597-2  -1.512-5 | 1.033+0  -1.698-2  3.177-5 | -1.396+0  5.306-3  -3.450-7 | 8.827-1  -8.470-4  -4.898-6 | -2.992-1  1.486-3 | 6.744-1  -2.717-4 | -4.625-1  -2.143-4 | -4.482-2  -4.143-4 | -6.840-3  5.371-5 |
| Solid, H (*m*) | 0.211 | 0.275 | -0.012 | 1.56 | 0 | -1.309-3 | 1.034+0 | -5.580-1 | 2.393+0 | -2.952+0 | 1.624+0 | -3.319-1 |  |  |  |
| Solid, H (*f*) | 0.049 | 0.076 | -0.013 | 1.50 | 0  10 | 6.154-4  2.399+0 | 9.859-1  -1.300+0 | 1.243-1  -6.413-1 | 3.883+0  2.390-1 | -1.336+1  3.764-1 | 1.817+1  -2.497-1 | -9.051+0  4.704-2 | -1.340-1 | -6.057-1 | 4.527-1 |
| Solid, N (*m*+*f*) | 0 | 0 | 0 | 1.61 | 0  10  20 | 1.371-3  -1.021-1  2.107-3 | 9.634-1  2.123-1 | 1.797+0  -1.480-1 | -4.507+0  1.080-2 | 4.454+0  2.591-2 | -2.018+0  -1.446-1 | 2.482+0  9.808-2 | -1.980+0  -2.482-3 | -9.639-1  -8.305-4 | 1.138+0  -7.276-3 |
| Solid, N (*m*) | 0.070 | 0.105 | -0.030 | 1.22 | 0 | 3.436-3 | 8.111-1 | 1.971+0 | -4.622+0 | 2.589+0 | 4.916+0 | -6.300+0 | -2.371-1 | 3.169+0 | -1.158+0 |
| Solid, N (*f*) | 0 | 0 | 0 | 1.35 | 0  10 | -5.760-4  -3.639-1 | 1.150+0  -8.108-1 | 1.243+0  3.961-1 | -4.819+0 | 8.959+0 | -6.302+0 | 1.265+0 | -2.020+0 | 2.346+0 | 4.348-1 |

(2) Liquid cancer in Hiroshima (H) and Nagasaki (N) (Mortality)

| City and gender | *t*_1_ (Sv) | *t*_2_ (Sv) | μ | *x*_u_(Sv) | *k* | *θ*_k+0_ | *θ*_k+1_ | *θ*_k+2_ | *θ*_k+3_ | *θ*_k+4_ | *θ*_k+5_ | *θ*_k+6_ | *θ*_k+7_ | *θ*_k+8_ | *θ*_k+9_ |
| --- | --- | --- | --- | --- | --- | --- | --- | --- | --- | --- | --- | --- | --- | --- | --- |
| Liquid, H (*m*+*f*) | 0.232 | 0.316 | -0.166 | 2.45 | 0 | -4.390-3 | 9.081-1 | -2.148-1 | 1.661+0 | -1.601+0 | 6.321-1 | 6.640-2 | -1.055-1 | 1.936-2 |  |
| Liquid, H (*m*) | 0.178 | 0.271 | -0.105 | 1.73 | 0 | 1.426-2 | 5.534-1 | 1.729+0 | -5.483-1 | -1.585+0 | 1.912+0 | -2.745-1 | -3.559-1 | 1.118-1 |  |
| Liquid, H (*f*) | 0.239 | 0.598 | -0.190 | 1.89 | 0  10 | 1.098-2  -6.610-2 | 5.436-1 | 1.290+0 | -9.613-1 | -9.843-1 | 2.043+0 | -2.760-1 | -5.349-1 | -1.096-1 | 2.696-1 |
| Liquid, N (*m*+*f*) | ---* | --- | --- | 2.35 | 0  10 | 7.087-2  -5.462-2 | -4.784-1 | 8.162+0 | -1.533+1 | 1.186+1 | -3.811+0 | 1.301+0 | -6.826-1 | -2.041-1 | 2.710-1 |
| Liquid, N (*m*) | --- | --- | --- | 1.83 | 0  10  20 | 1.539-2  -1.715-1  -3.580-4 | 8.627-1  5.839-2 | 1.830-1  1.369-2 | 4.841-1  2.096-3 | -8.374-1  3.811-3 | -3.217-2  4.060-3 | 9.588-1  1.817-3 | -5.682-1  -1.499-4 | 3.346-1  -1.109-3 | -1.359-1  -4.995-4 |
| Liquid, N (*f*) | --- | --- | --- | 1.76 | 0 | 2.803-2 | 2.088-1 | 6.244+0 | -1.621+1 | 1.777+1 | -9.059+0 | 1.938+0 | -8.619-2 |  |  |
| Liquid,  N-nonFW (*m*+*f*)** | --- | --- | --- | 1.52 | 0  10 | 8.215-3  8.995-2 | 8.579-1  -1.092+0 | 1.254+0  3.495-1 | -3.247+0 | 3.319+0 | -9.022-1 | 6.086-2 | -1.017+0 | -9.078-1 | 2.148+0 |
| Liquid,  N-nonFW (*m*)** | --- | --- | --- | 1.52 | 0  10 | 1.982-2  -7.981-1 | 6.051-1  1.924-1 | 3.220+0 | -7.755+0 | 6.666+0 | 1.321+0 | -5.317+0 | 1.130+0 | 1.319+0 | 3.327-1 |
| Liquid,  N-nonFW (*f*)** | --- | --- | --- | 1.52 | 0  10  20 | 2.254-2  8.348-1  1.422-2 | 3.999-1  -1.678-3  -3.931-3 | 4.351+0  2.533-1  2.384-3 | -1.106+1  2.470-1  -1.026-2 | 1.252+1  -1.040-1  -1.192-3 | -4.605+0  -3.209-1  2.265-3 | -1.025+0  2.951-3 | -9.112-2  4.484-3 | 6.872-1  -3.350-3 | -1.122-1  5.073-2 |

*) Dashed lines: Threshold cannot be determined due to the presence of a transient rise of *ERR* at low doses. **) Nagasaki cohort excluding factory workers (FW).

(3) Solid cancer by site in Hiroshima (H) (Incidence)

| Site and gender | *t* _1_(Sv) | *t*_2_ (Sv) | μ | *x*_u_(Sv) | *k* | *θ*_k+0_ | *θ*_k+1_ | *θ*_k+2_ | *θ*_k+3_ | *θ*_k+4_ | *θ*_k+5_ | *θ*_k+6_ | *θ*_k+7_ | *θ*_k+8_ | *θ*_k+9_ |
| --- | --- | --- | --- | --- | --- | --- | --- | --- | --- | --- | --- | --- | --- | --- | --- |
| Oral, H(*m*) | 0 | 0 | 0 | 1.56 | 0 | -8.770-3 | 1.220+0 | -3.019-3 | 1.475+0 | -1.742+0 | 6.062-2 | 8.049-1 | -2.987-1 |  |  |
| Oral, H(*f*) | 0.180 | 0.274 | -0.133 | 1.73 | 0  10  20  30 | 2.361-2  -7.359-2  -3.867-42.389-6 | 3.480-1  -4.568-2  1.404-4 | 2.830+0  1.724-2  1.434-4 | -3.456+0  -9.161-3  -6.999-5 | 1.705+03.986-3  -4.560-5 | -1.172+0  2.988-3  -2.326-5 | 4.991-1  6.052-4  9.217-7 | 4.314-1  3.210-4  -1.334-6 | 1.075-1  2.617-5  -7.382-6 | -1.173-1  9.321-4  2.218-6 |
| Esophageal, H(*m*) | 0.299 | 0.893 | -0.027 | 1.56 | 0 | -4.734-3 | 1.131+0 | -9.273-1 | 2.065+0 | -1.464+0 | 3.661-1 |  |  |  |  |
| Esophageal, H(*f*) | 0.126 | 0.245 | -0.245 | 1.73 | 0 | 4.960-2 | -6.709-1 | 9.397+0 | -1.644+1 | 1.407+1 | -5.753+0 | 9.276-1 |  |  |  |
| Stomach, H (*m*) | 0.211 | 0.339 | -0.035 | 1.56 | 0 | 3.849-3 | 8.961-1 | 1.981-1 | 4.191-1 | -5.057-1 | -5.436-1 | 6.216-1 | 4.140-1 | -5.962-1 | 1.638-1 |
| Stomach, H (*f*) | 0 | 0 | 0 | 1.73 | 0  10  20 | 3.053-3  -3.823-3  2.935-4 | 9.408-1  3.251-2  -7.023-5 | 3.070-1  -3.904-4  6.407-5 | 2.433-1  -5.937-4  1.077-4 | -7.432-1  -1.099-3  -2.428-5 | 5.730-1  4.918-4  -1.847-5 | 1.926-1  -2.455-3 | -3.834-1  -2.354-4 | 1.654-1  -7.106-5 | 4.070-2  -3.958-4 |
| Colorectal, H(*m*) | 0 | 0 | 0 | 1.56 | 0 | -1.033-4 | 1.052+0 | 8.186-1 | -5.949-1 | 1.743-1 |  |  |  |  |  |
| Colorectal, H(*f*) | 0 | 0 | 0 | 1.73 | 0  10 | -4.114-3  1.299-2 | 9.457-1 | 1.866+0 | -4.668+0 | 3.951+0 | -7.328-1 | 8.773-1 | -2.193+0 | 1.455+0 | -3.329-1 |
| Pancreas, H (*m*) | 0.760 | 1.101 | -0.180 | 1.56 | 0  10 | -2.897-2  7.536-1 | 2.329+0 | -7.528+0 | 1.307+1 | -5.747+0 | -7.087+0 | 6.121+0 | 0.052+0 | -3.928-2 | -1.979+0 |
| Pancreas, H (*f*) | 0.056 | 0.102 | -0.044 | 1.73 | 0  10 | -9.672-3  -4.646-3 | 1.096+0  -3.730-2 | 1.170-1 | 1.116+0 | -4.326+0 | 5.052+0 | -9.005-1 | -1.220+0 | 2.004-2 | 3.646-1 |
| Gallbladder, H (*m*) | 0.679 | 1.258 | -0.258 | 1.56 | 0  10 | -8.268-3  3.705-2 | 6.848-1  2.283-2 | 1.006+0 | -4.307+0 | 5.718+0 | -9.260-2 | -3.981+0 | 1.347+0 | 1.154+0 | -6.941-1 |
| Gallbladder, H (*f*) | 0.525 | 0.901 | -0.105 | 1.73 | 0  10 | -1.455-2  -1.114-1 | 1.403+0  1.278-1 | -2.029+0 | 6.408-1 | 5.983+0 | -5.811+0 | -2.703+0 | 4.398+0 | 4.746-4 | 8.552-1 |
| Liver, H (*m*) | 0.264 | 0.334 | -0.031 | 1.56 | 0 | -5.740-3 | 1.086+0 | -9.419-1 | 2.813+0 | -1.451+0 | -6.351-1 | 4.623-1 | 1.527-1 | -9.442-2 |  |
| Liver, H (*f*) | 0.121 | 0.327 | -0.131 | 1.73 | 0  10 | -1.161-3  5.779-2 | 8.014-1  -5.543-2 | 7.838-1  1.513-2 | -2.563-1  4.550-2 | -8.671-1  6.142-3 | 1.298-1  -1.161-3 | 7.222-1  -1.155-3 | -6.795-2 | -1.170-1 | -7.753-2 |
| Lung, H (*m*) | 0.240 | 0.477 | -0.072 | 1.56 | 0  10  20 | 3.981-3  -4.130-1  -8.179-3 | 9.068-1  2.794-1  -2.333-3 | -4.102-2  -5.524-1  1.092-3 | -4.931-1  1.373-1  8.671-4 | 4.755+0  6.373-2  -1.448-4 | -7.573+0  -1.988-2  1.520-3 | 4.174+0  -6.237-3  -1.545-4 | -1.340+0  9.574-2  -2.792-4 | 1.303+0  -1.423-2 | -3.717-2  -2.187-2 |
| Lung, H (*f*) | 0 | 0 | 0 | 1.73 | 0  10 | 1.133-2  5.860-2 | 7.594-1  1.009-1 | 3.158+0  -7.046-3 | -5.729+0  -2.143-2 | 4.692+0  1.261-2 | -1.194+0  -5.772-3 | 3.470-1  -6.127-4 | -1.985-1  -1.710-3 | -1.536-1  7.826-4 | -1.206-1  1.938-4 |
| Thyroid, H (*m*) | 0 | 0 | 0 | 1.56 | 0 | -1.137-2 | 1.714+0 | -1.192+0 | -7.725+0 | 3.284+1 | -4.380+1 | 2.512+1 | -5.304+0 |  |  |
| Thyroid, H (*f*) | 0 | 0 | 0 | 1.73 | 0 | -2.002-3 | 1.137+0 | 1.564-1 | 7.110+0 | -8.928+0 | -3.414+0 | 1.464+1 | -1.192+1 | 4.238+0 | -5.807-1 |
| Urinary, H(*m*) | 0.466 | 0.886 | -0.191 | 1.03 | 0  10 | -1.542-2  -1.839+1 | 1.280+0  7.118+0 | -3.052+0 | 5.089+0 | 1.954+0 | -7.282+0 | 3.160-1 | -1.150+0 | 5.222+0 | 9.970+0 |
| Urinary, H(*f*) | 0 | 0 | 0 | 1.18 | 0  10 | 2.712-2-7.797+0 | 6.101-1  -2.439-2 | 3.739+0  8.420-1 | -5.888+0  -1.275+0 | 8.081+0  2.145+0 | -5.430+0  -1.406+0 | -5.536+0  1.389+0 | 5.381+0-6.401-1 | 1.800+0 | 5.811+0 |
| Skin, H(*m*) | 0.047 | 0.135 | -0.179 | 1.54 | 0  10 | -6.945-3  -2.618-1 | 9.891-1  2.503-1 | 6.118-1  2.537-1 | -2.175+0  1.552-2 | 3.071+0  -3.124-2 | 2.272-1  -3.389-2 | -1.741+0-5.055-2 | 4.075-2  -5.286-2 | 4.828-1  2.040-2 | -3.685-1 |
| Skin, H(*f*) | 0.289 | 0.561 | 0.123 | 1.18 | 0  10 | 1.643-25.695+1 | 1.440+0  -7.174+0 | -3.311+0  1.049+1 | 1.155+0  -4.496-1 | 3.623+1  -3.404+1 | -8.945+12.100+1 | 5.275+1  2.028+1 | 3.384+1  4.564+0 | 1.074+1  -2.058+1 | -9.350+1  9.634+0 |
| CN-system, H(*m*) | 0.206 | 0.430 | -0.280 | 1.56 | 0 | 5.616-3 | 8.314-1 | -1.762+0 | 7.698+0 | -7.692+0 | 3.840+0 | -7.952-1 |  |  |  |
| CN-system, H(*f*) | 0 | 0 | 0 | 1.73 | 0  10  20  30 | -1.094-2  -2.317-1-3.100-4  8.006-7 | 1.394+0  -3.000-1  3.660-4 | 8.965-1  1.542-1  -2.484-4 | -2.546+0  1.749-2  8.987-5 | 4.141+0  -4.357-2  -1.841-5 | -1.665+0  -4.542-3  -6.698-5 | -2.197+0  1.497-2  -1.640-5 | 1.067+0  -2.943-3  1.692-5 | 6.717-1  1.695-3  -7.177-6 | 2.507-1  6.682-4  3.637-6 |
| Prostate, H (*m*) | 0.122 | 0.305 | -0.193 | 1.56 | 0 | 6.974-3 | 5.240-1 | 2.523+0 | -4.754+0 | 5.057+0 | -2.838+0 | 6.264-1 |  |  |  |
| Breast, H (*f*) | 0.107 | 0.133 | -0.022 | 1.73 | 0  10 | 8.484-3  7.787-3 | 7.062-1  5.610-2 | 2.000+0  -3.329-2 | -1.488+0  -2.222-2 | -1.182-1  -1.577-3 | 1.390+0  1.381-2 | -5.511-1  -3.769-3 | -3.094-1 | 6.444-2 | 7.726-2 |
| Cervix, H (*f*) | 0 | 0 | 0 | 1.73 | 0 | -1.174-3 | 1.003+0 | -4.750-1 | 1.509+0 | -1.804+0 | 9.276-1 | -1.731-1 |  |  |  |
| Ovarian, H(*f*) | 0.117 | 0.178 | -0.103 | 1.73 | 0  10 | 1.299-2  1.981-1 | 3.096-1 | 5.862+0 | -1.594+1 | 1.929+1 | -8.261+0 | -2.133+0 | 2.197+0 | 6.607-1 | -8.833-1 |
