# Supplementary material for: Cancer risk at low doses of ionizing radiation: artificial neural networks inference from atomic bomb survivors
Source: J Radiat Res. 2013 Dec 22;55(3):391–406. doi: 10.1093/jrr/rrt133 (PMC4014156; doi:10.1093/jrr/rrt133)
Supplement: Supplementary Data [file supp_rrt133_rrt133supp_fig.docx]

**Supplementary Figure**

**Cancer risk at low doses of ionizing radiation: Artificial neural networks inference from atomic bomb survivors**

Masao S. SASAKI, Akira TACHIBANA and Shunichi TAKEDA

[J. Radiat. Res., ***:***-***,****]


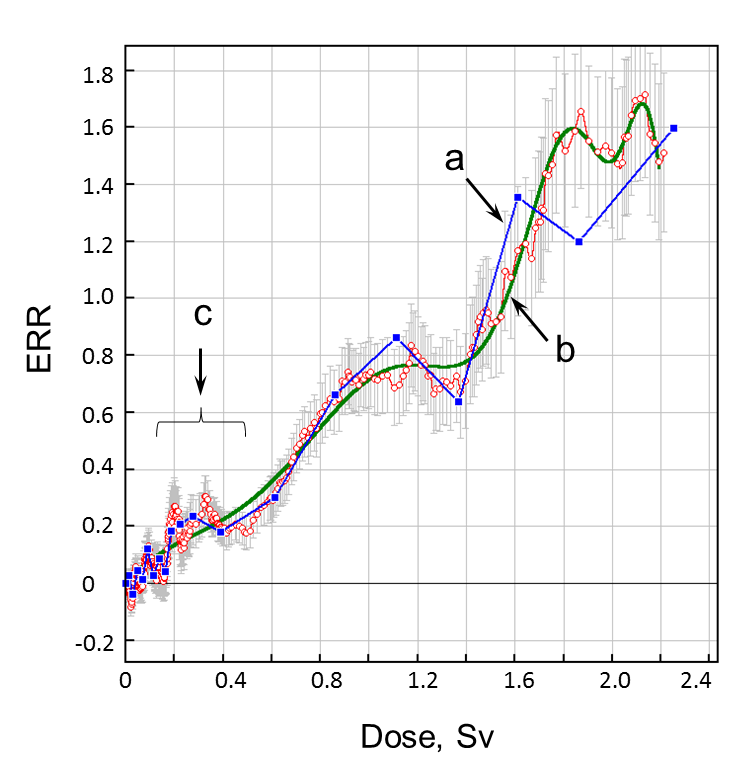


**Fig. S1. Cross-validation of methods:** Comparison of conventional piecewise dose-category method and the ANN method used in the present study o calculate the *ERR* of solid cancer incidence in a combined Hiroshima and Nagasaki cohort. To compare across the same dosimetry platform, all calculations were made with the colon dose, with neutrons weighted using an RBE=10. The *ERR* values obtained by the piecewise dose-category method (20 dose groups) as previously adopted by Preston *et al*. [Radiat Res 2007;**168**:1-64] are shown by solid squares connected by thick straight lines (**a**). The thick curve (**b**) shows the probability density function of *ERR* calculated by the ANN method. An abnormal elevation of *ERR* at low doses is shown by an arrow (**c**). Observed *ERR* data points obtained by moving window averaging w1500s50 are presented by open circles with an 80 % C.I.
